# Supplementary figures and images for: Comparative Metagenomics and Metabolomes Reveals Abnormal Metabolism Activity Is Associated with Gut Microbiota in Alzheimer’s Disease Mice
Source: Int J Mol Sci. 2022 Sep 30;23(19):11560. doi: 10.3390/ijms231911560 (PMC9569518; doi:10.3390/ijms231911560)

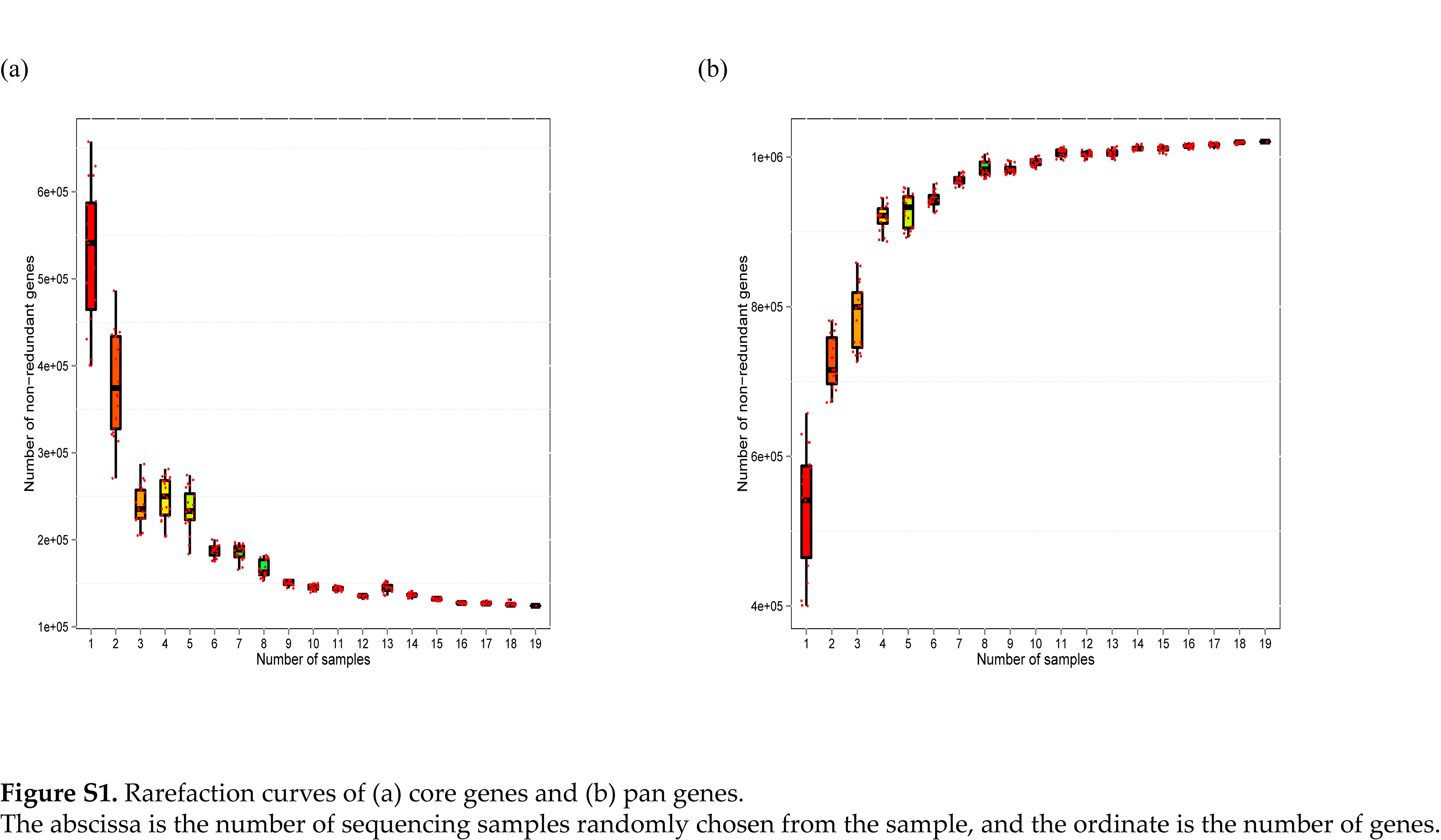

Supplement: Supplementary file 1 [file ijms-23-11560-s001.zip › Supplementary Files/Figures/Figure S1.jpg]

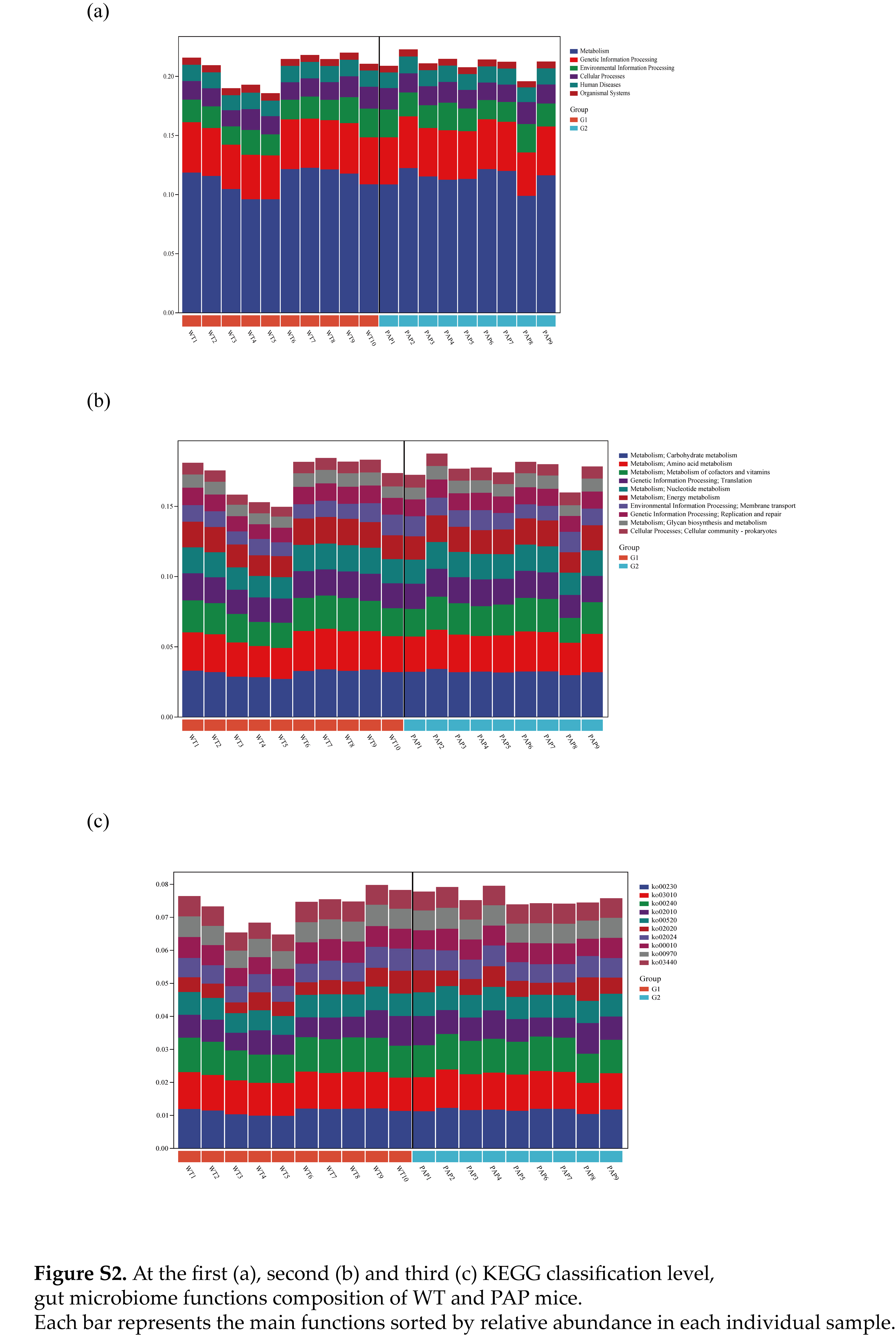

Supplement: Supplementary file 1 [file ijms-23-11560-s001.zip › Supplementary Files/Figures/Figure S2.jpg]

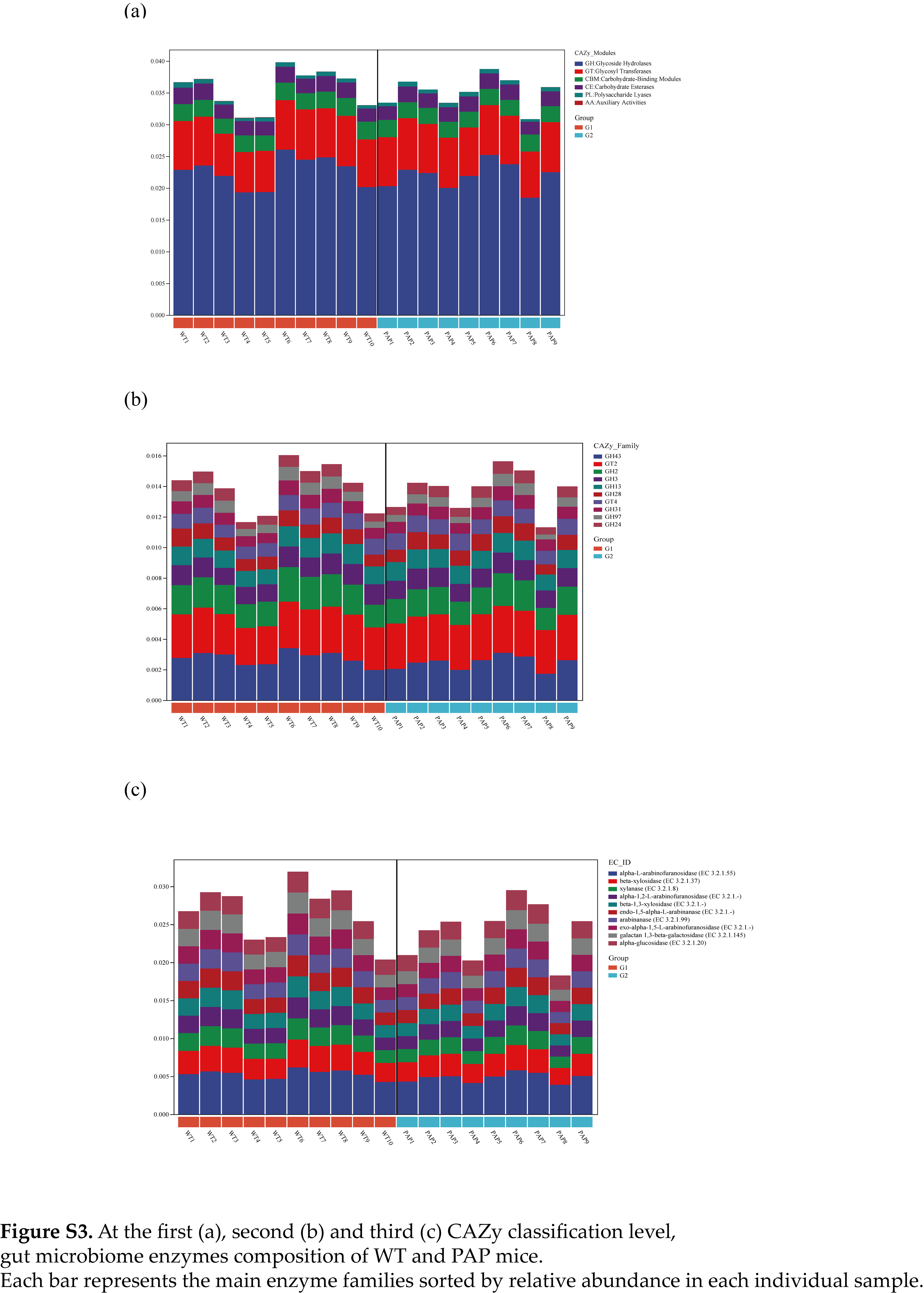

Supplement: Supplementary file 1 [file ijms-23-11560-s001.zip › Supplementary Files/Figures/Figure S3.jpg]

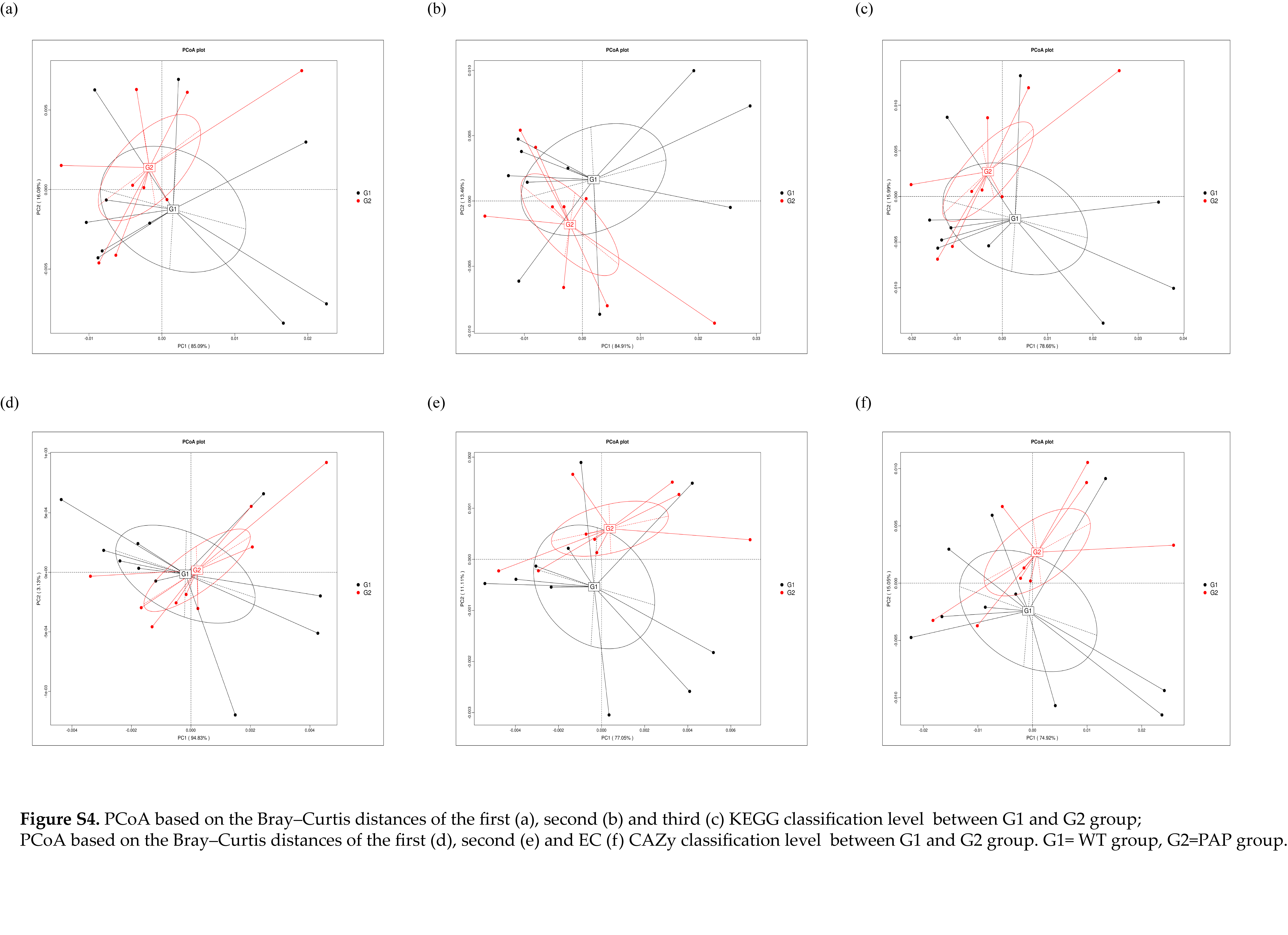

Supplement: Supplementary file 1 [file ijms-23-11560-s001.zip › Supplementary Files/Figures/Figure S4.jpg]
